# Metagenomic Insights in Antimicrobial Resistance Threats in Sludge from Aerobic and Anaerobic Membrane Bioreactors

**DOI:** 10.1021/acs.est.4c10879

**Published:** 2025-03-12

**Authors:** Julie Sanchez Medina, Shuo Zhang, Shaman Narayanasamy, Changzhi Wang, Bothayna Al-Gashgari, Pei-Ying Hong

**Affiliations:** † Environmental Science and Engineering Program, Biological and Environmental Science and Engineering Division, King Abdullah University of Science and Technology (KAUST), Thuwal 23955-6900, Kingdom of Saudi Arabia; ‡ Center of Excellence on Sustainable Food Security, King Abdullah University of Science and Technology (KAUST), Thuwal 23955-6900, Saudi Arabia; § Bioengineering Program, Biological and Environmental Science and Engineering Division, King Abdullah University of Science and Technology (KAUST), Thuwal 23955-6900, Kingdom of Saudi Arabia; ∥ Bioscience Program, Biological and Environmental Science and Engineering Division, King Abdullah University of Science and Technology (KAUST), Thuwal 23955-6900, Kingdom of Saudi Arabia

**Keywords:** antibiotic resistance genes, sludge, horizontal gene transfer, metagenomics, membrane bioreactor

## Abstract

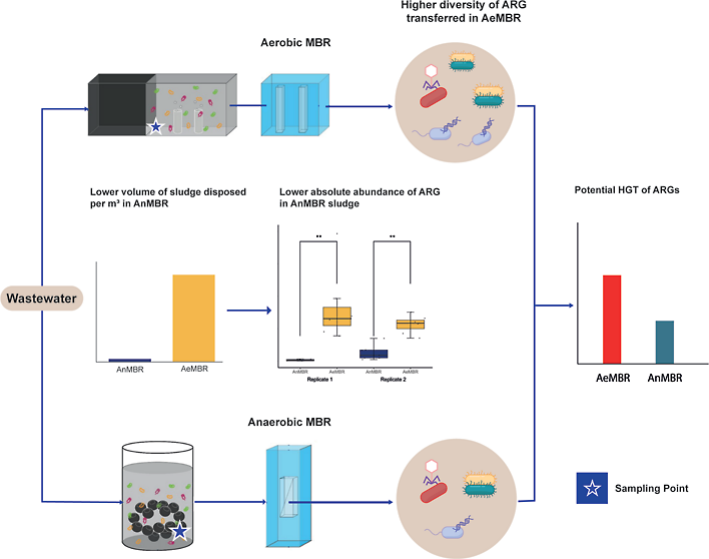

Sludge is a biohazardous solid waste that is produced during wastewater treatment. It contains antibiotic resistance genes (ARGs) that pose significant antimicrobial resistance (AMR) threats. Herein, aerobic and anaerobic membrane bioreactors (AeMBRs and AnMBRs, respectively) were compared in terms of the volume of waste sludge generated by them, the presence of ARGs in the sludge, and the potential for horizontal gene transfer (HGT) events using metagenomics to determine which treatment process can better address AMR concerns associated with the generation of waste sludge. The estimated abundance of ARGs in the suspended sludge generated by the AnMBR per treated volume is, on average, 5–55 times lower than that of sludge generated by the AeMBR. Additionally, the ratio of potential HGT in the two independent runs was lower in the anaerobic sludge (0.6 and 0.9) compared with that in the aerobic sludge (2.4 and 1.6). The AnMBR sludge exhibited reduced HGT of ARGs involving potential opportunistic pathogens (0.09) compared with the AeMBR sludge (0.27). Conversely, the AeMBR sludge displayed higher diversity and more transfer events, encompassing genes that confer resistance to quinolones, rifamycin, multidrug, aminoglycosides, and tetracycline. A significant portion of these ARGs were transferred to *Burkholderia* sp. By contrast, the AnMBR showed a lower abundance of mobile genetic elements associated with conjugation and exhibited less favorable conditions for natural transformation. Our findings suggest that the risk of potential HGT to opportunistic pathogens is greater in the AeMBR sludge than in AnMBR sludge.

## Abstract 2

This study indicates a reduced risk of horizontal gene transfer of antibiotic resistance genes in the sludge of anaerobic membrane bioreactors. The anaerobic process also generates a reduced volume of sludge, suggesting a diminished threat of antimicrobial resistance.

## 1 Introduction

Antibiotic resistance, often referred to as a silent pandemic, is estimated to have contributed to ∼4.95 million deaths in 2019.^1^ The factors leading to antibiotic resistance extend beyond the clinical environment and should be viewed through a One-Health perspective, which highlights the interconnectedness of human and animal health as well as their shared environment.^2^ Within this shared environment, conventional activated sludge-based wastewater treatment plants (WWTPs) serve as a significant source for the dissemination of antibiotic resistance genes (ARGs).^3−5^ This occurs because of the high cell density in the bioreactor along with the selective pressure exerted by antibiotics, disinfectants, and other pollutants in the sewage, which facilitates the emergence and coselection of ARGs during the treatment processes.^6^

Compared with conventional activated sludge processes, membrane bioreactors (MBRs) are better at removing emerging contaminants, including antibiotic-resistant bacteria (ARB) and ARGs from sewage, because of the additional barrier created by membrane filtration.^7,8^ MBRs can be operated in either aerobic (AeMBR) or anaerobic (AnMBR) modes. Although AeMBRs are more commonly used in full-scale WWTPs than AnMBRs, the latter offers advantage of lower energy costs and reduced solid waste disposal requirements.^9,10^ Additionally, the operation of an AnMBR typically involves a longer solid retention time (SRT), which may correlate with decreased ARGs abundance in anaerobic systems, compared with that of an AeMBR.^11^ The lower volume of sludge generated by AnMBRs than AeMBRs may also contribute to a reduced risk of antimicrobial resistance associated with the generated solid waste.^12^ This is primarily because sludge offers conditions that promote the spread of ARGs through horizontal gene transfer (HGT).^13^ Furthermore, previous studies have reported that a typical sludge sample contains between 10^9^ and 10^11^ copies of total ARGs per gram.^12,14^ Therefore, the waste sludge produced by WWTPs serves as a significant reservoir of ARGs and ARBs, both of which can have potentially harmful environmental impacts following sludge disposal.^15,16^ This concern is particularly pronounced in communities that opt for the direct landfill of solid waste without digestion or any other form of sludge stabilization protocols.

Herein, we collected sludge samples from an AeMBR and AnMBR, each treating the same source of untreated wastewater. We used metagenomics to examine the differences in the ARGs present in the sludge produced by the two systems and the potential occurrence of HGT among them. This study aimed to determine which MBR configuration most effectively minimized the risks associated with antimicrobial resistance (AMR) in sludge waste. Specifically, we investigated whether the diversity and relative abundance of ARGs in the sludge, as well as the extent of HGT, differed based on the wastewater treatment process. This understanding can aid in selecting the appropriate treatment technology to enhance the mitigation of AMR threats linked to wastewater management.

## 2 Materials and Methods

### 2.1 MBR Operating Conditions

The AeMBR evaluated in this study functions as the primary biological treatment process within a decentralized full-scale WWTP located at KAUST, Saudi Arabia. The operating conditions have been described elsewhere.^17^

In summary, the AeMBR includes a primary clarifier followed by anoxic and aerobic-activated sludge tanks, along with a submerged membrane tank. The SRT is maintained at 40 days, and waste sludge generation is approximately 0.62 L per m^3^ of treated wastewater. In parallel, the AnMBR operates as a pilot-scale reactor on the KAUST WWTP premises, receiving the same influent stream as the AeMBR, positioned downstream from the primary clarifier. The AnMBR features an attached growth configuration, capable of treating up to 108 L/d, with a SRT of 680 days. It generates minimal waste sludge, averaging just 0.001 L per m^3^ of treated wastewater. Both reactors maintain an average temperature of 30 °C and a pH of 7.2. Further reactor specifications related to the AnMBR have been described previously.^18^

The study was conducted with two biologically independent replicates, each involving experiments over two distinct time periods. The first period, from July 2021 to September 2021, featured the AnMBR operating at a hydraulic retention time (HRT) of 10 h, which is considered optimal for achieving efficient removal of organic matter and bacterial pathogens.^19^ The second period, from September 2021 to November 2021, saw the AnMBR operated at an HRT of 8 h, aligning it with the AeMBR, which was consistently maintained at an 8 h HRT during both durations.

### 2.2 Reactor Performance Evaluation

Chemical oxygen demand (COD), nitrite, nitrate, ammonia, and phosphate were measured using kits: LCK 314,341, 339, Nitrogen-Ammonia, Salicylate TNT Method 69 and LCK 348 (HACH UK). Mixed liquor suspended solids (MLSS) were measured according to APHA standard methods.^20^ Biogas production in the AnMBR and flow cytometry-based total bacterial counts (SYBR green I) were performed as described elsewhere.^19^

### 2.3 Sample Collection and DNA Extraction

For each of the two biologically independent experiments, samples were collected weekly for 8 weeks. At each designated time point, 50 mL influent and 50 mL suspended sludge were collected from both the aerobic and anaerobic MBRs for DNA isolation. The influent and suspended sludge samples were centrifuged for 10 min at 7500*g*, and the pellets were stored at −80 °C before DNA extraction using the DNeasy PowerLyzer Microbial kit (Qiagen, Hilden, Germany). Samples were pretreated as described previously.^21^ Extracted DNA was quantified using the Qubit dsDNA BR assay kit (Thermo Fisher Scientific, Waltham, MA, US).

### 2.4 16S rRNA Gene-Based Amplicon Sequencing

The V4–V5 region of the 16S rRNA genes was PCR-amplified using primers 515F (5′-Illumina overhang-GTGYCAGCMGCCGCGGTAA-3′) and 907R (5′-Illumina overhang-CCCCGYCAATTCMTTTRAGT-3′).^22^ Amplicons were purified using an AMPure XP bead kit (Beckman Coulter), then the Nextera XT Index kit (Illumina) was used for library preparation. Samples were normalized, pooled, and sequenced on the Illumina MiSeq platform. DNA sequences were processed using QIIME 2.^23^ After index removal, DADA2 was used for sequences denoising and chimera removal.^24^ Amplicon sequence variants were generated and classified using Silva SSU database version 138.^25^ Rarefaction was performed to equalize the total number of reads in all samples to match the sample with the lowest number of reads (*n* = 37,700). Beta diversity was assessed using the Bray–Curtis dissimilarity index and metric multidimensional plots.

### 2.5 Shotgun Metagenomics

DNA samples (100 ng) were processed individually using the TruSeq DNA library kit and sequenced on the Illumina NovaSeq6000 platform in the KAUST Bioscience Core Lab (150-bp paired-end reads). The average yield per sample was 60 million reads.

### 2.6 Resistome and Mobile Genetic Element Analyses

ARGs were identified and quantified using the ARG-OAP v.3 pipeline, with the SARG database as a ref (26). The relative abundance of the resistome was determined by calculating the number of reads of ARGs per total number of reads for each sample. The estimated ARGs abundance in copy number, scaled by the sludge disposal per treated volume, was calculated using the MLSS and sludge volume utilized in the DNA extraction, with the copy number of the ARGs being calculated as described in Text S1 using the following equation
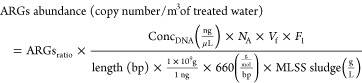

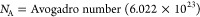

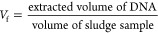

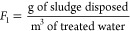

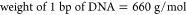


The estimated abundance of ARGs and the alpha and beta diversity of these genes were assessed using Primer-E v7.^27^ This analysis involved using the normalized copies of ARGs per copy of 16S rRNA sourced from the SARG database within the ARG–OAP pipeline. The Bray–Curtis dissimilarity index was used for the beta diversity assessment and metric multidimensional scaling plots were created.

Additionally, mobile genetic elements (MGEs) were identified and quantified by analyzing the normalized gene copies per copy of 16S rRNA using the ARG–OAP v.3 pipeline.^26^ This assessment relied on a custom, manually curated MGE database, mobile-OGdb, as a ref (28).

### 2.7 Assembly of Metagenome Samples and Identification of Metagenome-Assembled Genomes

The preprocessing of reads involved quality-based trimming using fastp with default parameters.^29^ MetaSPAdes was subsequently used for sequence assembly, and the relative abundance of contigs was calculated using CoverM v.0.7.0 (https://github.com/wwood/CoverM). Metabat, Concoct, and Anvio were used for genome binning.^30−32^ The obtained metagenome-assembled genomes (MAGs) were refined using MetaWRAP,^33^ and their quality was evaluated using CheckM. MAGs with >75% completeness and <10% contamination were considered for further analysis. The taxonomic annotation of these MAGs was performed using GTDB-Tk.^34^

### 2.8 Identification of Potential Horizontal Gene Transfer

Potential HGT event analysis for the MAGs was performed using MetaCHIP at the taxonomical levels of order, family, and genus,^35^ as described in Text S2. The putative ARGs that were horizontally transferred were searched against the SARG database (v3.0) for ARGs using Diamond^36^ with BLASTx. A cutoff of 80% query cover and e-value of 1 × 10^–6^ was applied to retain high-quality hits for downstream analyses.

The proportion of potential HGT events per million reads was calculated to normalize the total potential HGT and the potential HGT events that involved ARGs. The classification of the donor and recipient MAGs was obtained from the GTDB-Tk results.^34^

### 2.9 Omics-Based Analysis to Characterize HGTs Related to Transduction and Conjugation

To further characterize whether the potential HGT events for ARGs transfer were related to transduction or conjugation, we first identified the contigs that correspond to viruses and plasmids. Virsorter2 and GeNomad were used with default parameters to identify viral contigs in the sludge sample assembly.^37,38^ The resulting contigs were processed with Cobra^39^ to increase the completeness of the viral genomes. Only viral contigs of at least 2000 bp and with one viral hallmark gene were considered. Then, CheckV was used to evaluate the quality of the obtained contigs,^40^ and the database of the International Committee on Taxonomy of Viruses (ICTV) was used to classify the viral contigs.^41^

Plasmid contigs from the assembly were also predicted using GeNomad. Only plasmid contigs with more than 2000 bp and with at least one hallmark gene were considered for the analysis. The ConjScan model was used to detect conjugative and mobilizable elements in the plasmid contigs.^42^

The contigs for plasmids and viruses were aligned with the SARG database (v3.0) using BlastX in Diamond^36^ to identify those that potentially contained ARGs with a query coverage of 80% and an *e*-value of 1 × 10^–6^. Following this, manual curation was conducted to pinpoint the contigs with transferred ARGs within the total viral and plasmid contigs extracted from the sludge samples obtained from the AeMBR and AnMBR. This process facilitates an estimation of the prevalence of possible HGT events, with particular emphasis on potential transduction or conjugation mechanisms. Further details about the methods used to compare the abundance of viral and plasmid contigs in the sludge can be found in Text S3.

### 2.10 Natural Transformation Assay with *Acinetobacter baylyi* ADP1

To evaluate whether sludge from the AnMBR and AeMBR systems can stimulate natural transformation, we introduced the supernatant fraction of suspended sludge from both systems to a reporter strain of *A. baylyi* ADP1. For further details on this strain, see Text S4. The characteristics of *A. baylyi* ADP1 and the assay conditions were described elsewhere.^43^ In brief, *A. baylyi* ADP1 was incubated in LB medium with the sludge supernatant (1×) and 2 μg donor DNA (which contains a functional promoter) for 24 h at 37 °C with shaking at 200 rpm. After incubation, the colonies formed on LB agar plates containing spectinomycin correspond to the transformed cells of *A. baylyi* ADP1. To determine the total population of cells, we counted all the colonies grown on LB agar without spectinomycin. The transformation frequency was calculated by normalizing the number of transformed *A. baylyi* ADP1 cells against the total cell count of *A. baylyi* ADP1.

### 2.11 Statistical Analysis

A *t*-test was used to evaluate the removal of COD and total bacterial cells to identify significant differences in average removal rates. An analysis of similarities (ANOSIM) assessed the beta diversity of bacterial communities and ARGs in AnMBR and AeMBR sludge. Spearman’s rank correlation coefficient test analyzed the relationship between MLSS, the average ratio of HGT events, and HGT events involving transfer of ARGs. A Mann–Whitney analysis compared the average proportion of potential HGT between AnMBR and AeMBR and the proportion of HGT events linked to plasmid or viral contigs. For the natural transformation assay, a *t*-test with Helm–Bonferroni correction was applied. The null hypothesis was typically rejected at a *p*-value below 0.05, indicating a 95% confidence interval. All analyses were conducted using R version 4.2 or Primer version 7. More details on the statistical tests can be found in Text S5.

### 2.12 Sequence Accession

All FASTQ files from the partial 16S rRNA gene-based amplification and shotgun metagenomics analyses are publicly available in the European Nucleotide Archive under accession number PRJEB69272.

## 3 Results

### 3.1 AeMBR and AnMBR Performance and Microbial Communities

An AnMBR and AeMBR were operated using the same municipal wastewater as influent (Figure S1). Throughout the study, both bioreactors demonstrated strong performance, with no significant differences observed in terms of COD removal or bacterial cell removal. In the first replicate, where the HRT was 10 h for the AnMBR and 8 h for the AeMBR, the AnMBR achieved a COD removal rate of 89 ± 2%, while the AeMBR achieved a slightly higher rate of 92 ± 2%. The removal of bacterial cells was also comparable, with the AnMBR showing a reduction of 2.0 ± 0.2 log and the AeMBR 2.1 ± 0.4 log (Table S1 and Figure S2).

When the HRT was standardized to 8 h for both bioreactors in the second replicate, the COD removal for the AnMBR decreased slightly to 86.1 ± 3% but remained similar to the AeMBR’s COD removal of 91.8 ± 1.7%. Similarly, the bacterial cell removal remained comparable, with the AnMBR at 2.3 ± 0.4 log and the AeMBR at 2.4 ± 0.5 log (Table S2 and Figure S2).

**Figure 1:**
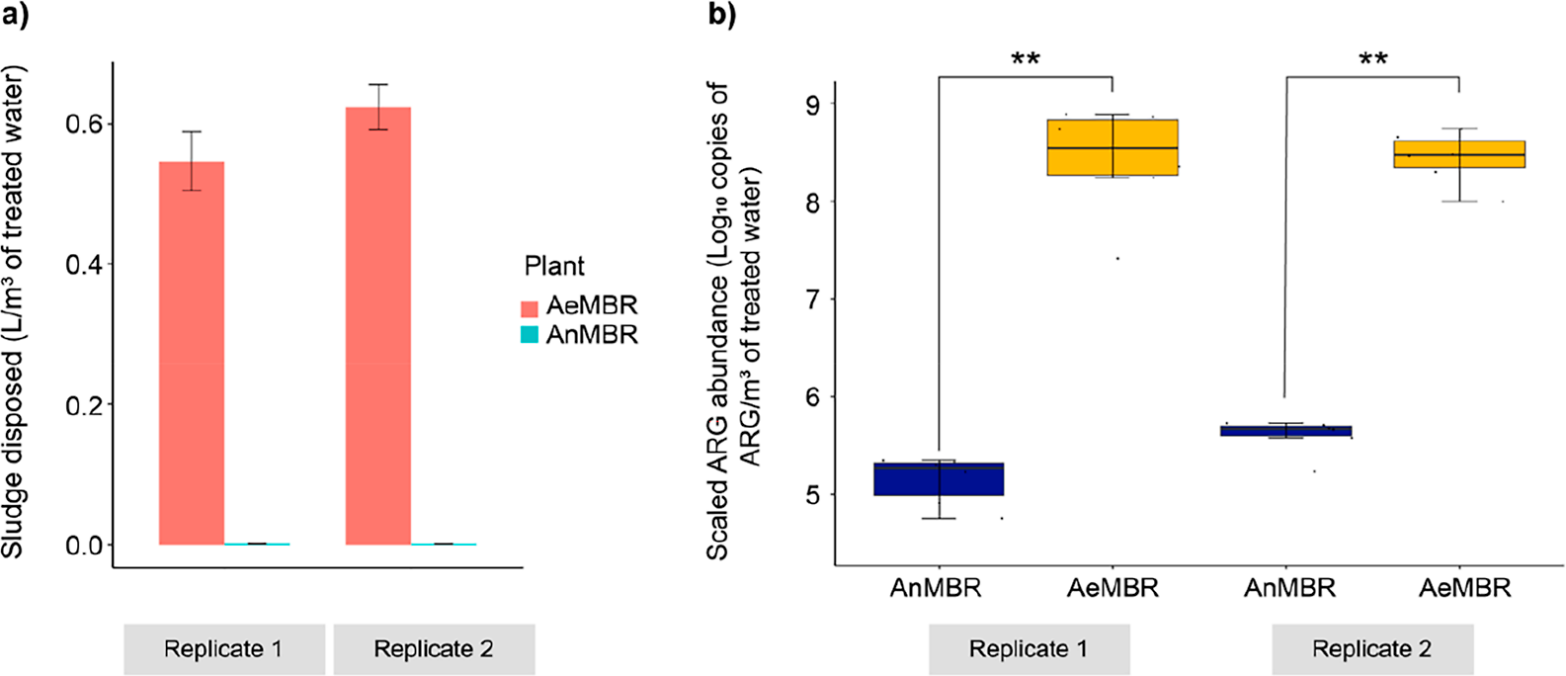
(a) Volume of sludge disposed from the AnMBR and AeMBR per m^3^ of treated wastewater. (b) Abundance of ARGs for the sludge of the AnMBR and AeMBR scaled by the sludge disposal per volume of treated wastewater reactor. **p-value lower than 0.01.

Biogas production in the AnMBR was consistent across both replicates, yielding 1.48 ± 0.2 L methane/day in the first replicate and 1.58 ± 0.4 L methane/day in the second replicate. Sludge production in the AeMBR averaged 0.62 L sludge/m^3^ of treated water, which was significantly more than that in AnMBR (0.001 L sludge/m^3^; Figure 1a). Thus, the sludge volume generated by the AnMBR was ∼60 times lower than that generated by the AeMBR.

**Figure 2:**
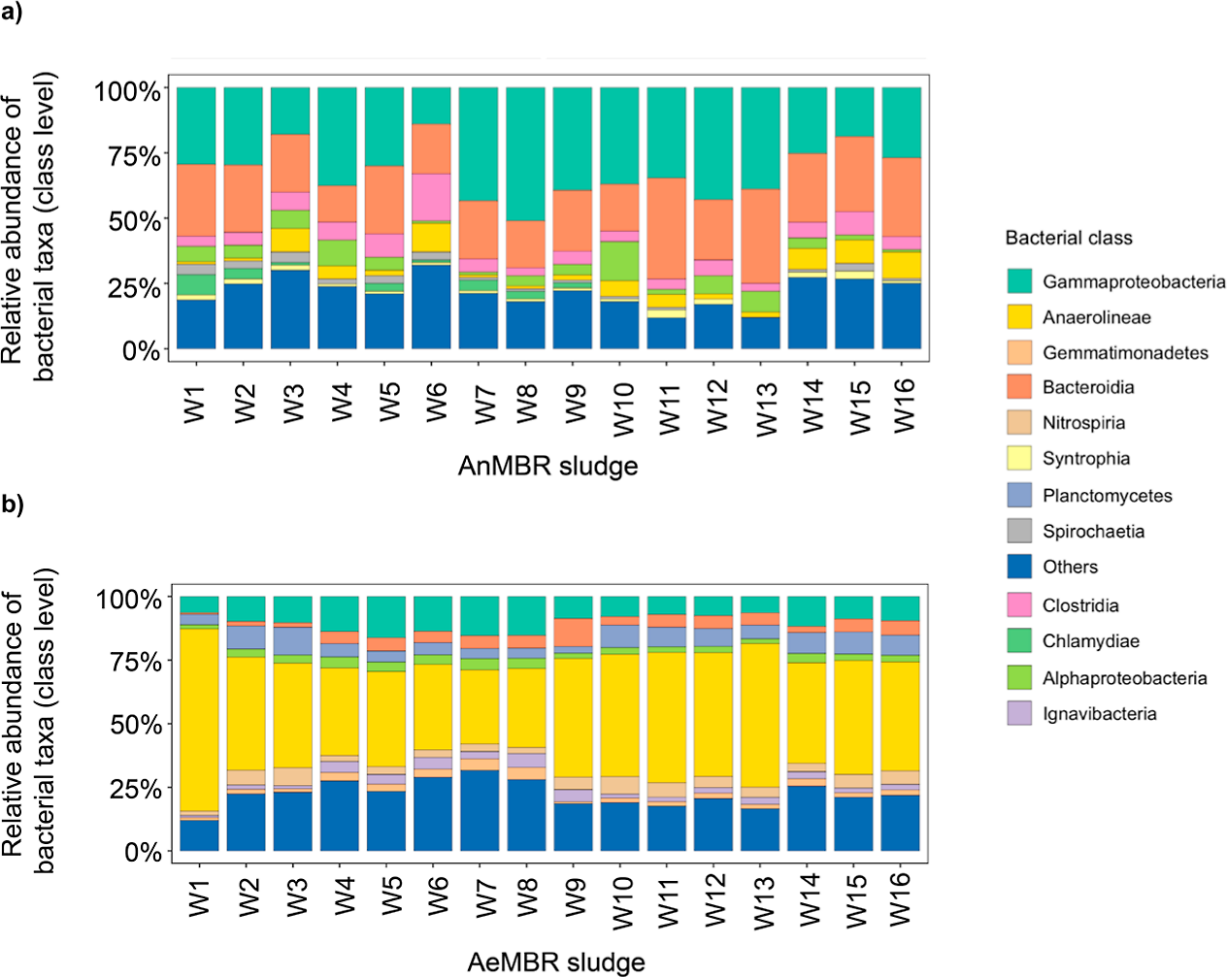
Microbial community profiles in both (a) AnMBR and (b) AeMBR sludge. Relative abundance of main bacterial groups at class level are shown for the AnMBR and AeMBR sludge, respectively.

In terms of microbial communities, both reactors maintained relatively stable compositions throughout the operation across both replicates. The predominant groups in the AnMBR sludge included Gammaproteobacteria (32% and 33%), Bacteroidia (22% and 28%), Clostridia (7% and 5%) and Anaerolineae (3.9% and 5.5%) (Figure 2a). Meanwhile, the AeMBR sludge predominantly consisted of Anaerolineae (40% and 47%), Gammaproteobacteria (13% and 9%), and Planctomycetes (6% and 7%; Figure 2b).

Furthermore, distinctive differences were noted in the beta diversity of the sludge from each bioreactor, indicating separate clusters for AnMBR and AeMBR sludge. The microbial community in AnMBR exhibited greater similarity to that in the influent across both the replicates (Figure S3). These observations were validated using an ANOSIM test, which showed a significant difference in microbial community compositions between the AnMBR and AeMBR sludge (*R* = 0.98 and *p*-value of 0.001 for replicate 1; *R* = 0.99 and *p*-value of 0.001 for replicate 2). The microbial community of the influent was significantly different from that of the AeMBR (*R* = 0.998 and *p*-value of 0.001 for replicate 1; *R* = 0.99 and *p*-value of 0.001 for replicate 2).

### 3.2 Differences in Relative ARGs Abundance and Diversity in AeMBR and AnMBR Sludge

We quantified the relative abundance of ARGs and assessed their beta diversity in the AnMBR and AeMBR sludge. Our goal was to determine whether the differences in microbial community composition between the two reactors were also reflected in the patterns of ARGs.

In replicate 1, the relative abundance of ARGs was significantly higher in the AnMBR sludge, measuring 6.3 × 10^–3^ ± 2.9 × 10^–3^% compared with 5.5 × 10^–4^ ± 1.4 × 10^–4^% in the AeMBR sludge (*p*-value = 0.0002). Similarly, replicate 2 exhibited a greater relative abundance of ARGs in the AnMBR sludge, which was 1.2 × 10^–2^% ± 8.2 × 10^–3^%, compared with 3.8 × 10^–4^% ± 1.0 × 10^–4^% in the AeMBR sludge (*p*-value = 0.0001). However, it is important to note that despite the lower relative abundance of ARGs in the AeMBR sludge, the larger sludge volume produced by the AeMBR (Section 3.1) suggests a higher estimated abundance of ARGs based on sludge disposal. In replicate 1, the estimated abundance of ARGs in the AnMBR sludge, factoring in sludge disposal, was 2 × 10^5^ ± 1.5 × 10^5^ copies of ARGs per m^3^ of treated wastewater. This was significantly lower than the 4.6 × 10^8^ ± 2.9 × 10^8^ copies of ARGs per m^3^ of treated wastewater in the AeMBR sludge (*p*-value = 0.0002). In replicate 2, the estimated absolute ARGs abundance in the AnMBR sludge was also lower than that in the AeMBR sludge (*p*-value = 0.00025, Table S3 and Figure 1b).

**Figure 3:**
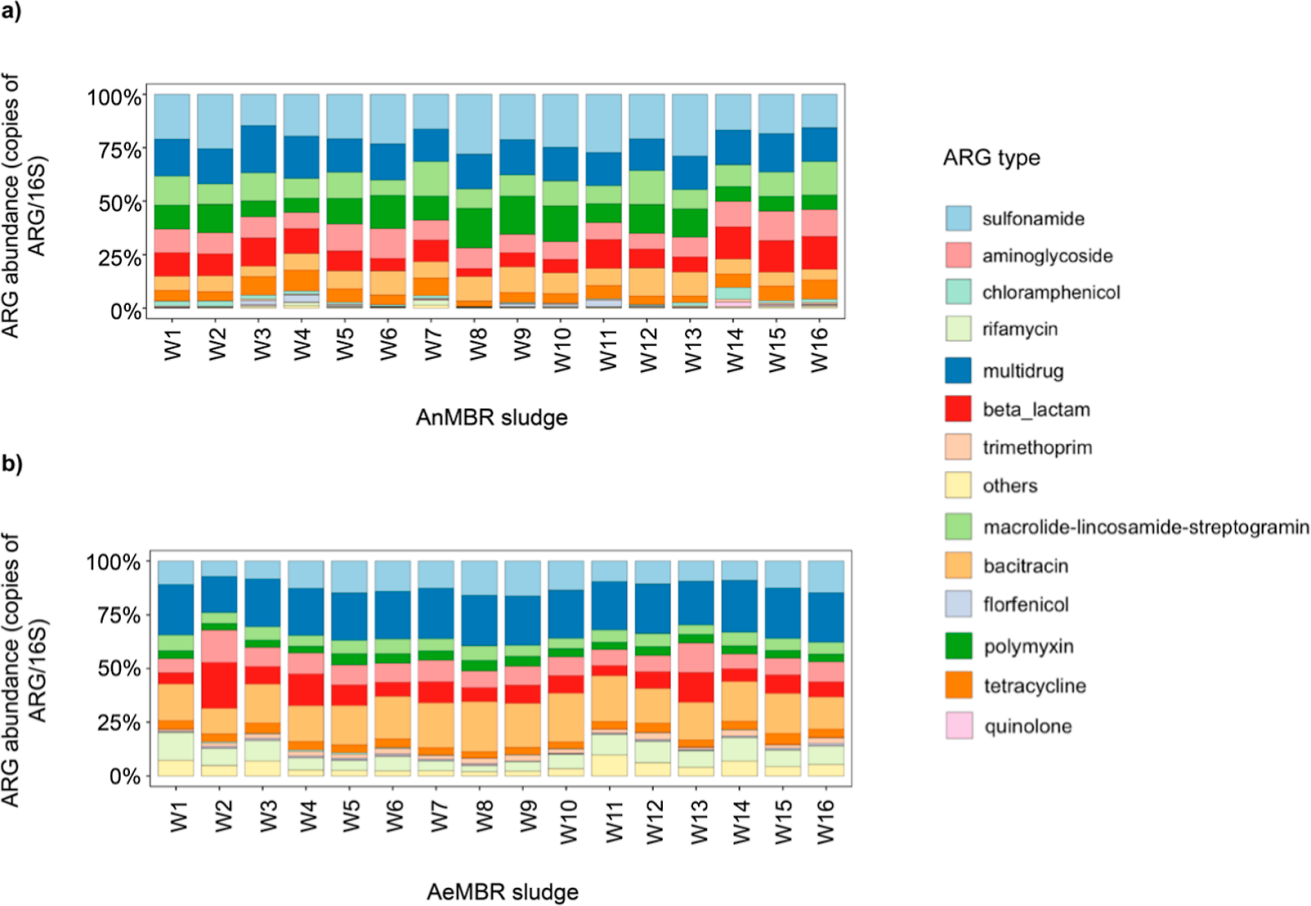
Relative abundance for (a) AnMBR and (b) AeMBR sludge for the main types of ARGs, respectively, normalized against the 16S rRNA genes.

Our analysis of the types of ARGs in the AnMBR sludge showed that the classes with the highest relative abundance corresponded to resistance against sulfonamides, multidrug, polymyxin, and macrolides. Specifically, these genes contributed to relative abundances of 22%, 16.5%, 11.6%, and 11.3%, respectively (Figure 3a). Notably, some of the most prevalent genes identified in the AnMBR sludge were *sul1* and *sul2* (sulfonamide resistance), *ugd* (polymyxin resistance), *qacE* (multidrug resistance), and *ereD* and *mefC* (macrolide resistance; Table 1). By contrast, the types of ARGs with the highest relative abundance in the AeMBR sludge corresponded to resistance to multidrug, bacitracin, sulfonamides, and beta-lactam, with respective abundances of 22%, 18.8%, 12.6%, and 9.3% (Figure 3b). The predominant genes in the AeMBR sludge were *qacE* for multidrug resistance, *bacA* for bacitracin resistance, *sul1* and *sul2* for sulfonamide resistance, and OXA-36 for beta-lactam resistance (Table 1). Interestingly, the ARGs composition in the AnMBR and AeMBR sludge remained relatively stable throughout the experiment. This stability implies that the modifications in the HRT for AnMBR did not significantly impact the ARGs composition.

**Table 1:** Most Abundant ARGs for AnMBR and AeMBR Sludge for Replicate 1 and Replicate 2 (Gene Copies Per Copy of 16S rRNA)

type/gene	AnMBR sludge		type/gene	AeMBR sludge	
	replicate 1	replicate 2		replicate 1	replicate 2
sulfonamide/*sul1*	0.02	0.025	multidrug/*qacE*	0.0036	0.0028
sulfonamide/*sul2*	0.007	0.01	bacitracin/*bacA*	0.0032	0.0022
multidrug/*qacE*	0.016	0.02	sulfonamide/*sul1*	0.0053	0.0056
polymyxin/*ugd*	0.046	0.053	sulfonamide/*sul2*	0.0024	0.002
macrolides/*ereD*	0.006	0.008	beta-lactam/OXA-36	0.0024	0.002
macrolides/*mefC*	0.004	0.006	polymyxin/*ugd*	0.04	0.041
bacitracin/*bacA*	0.009	0.01	macrolides/*ereD*	0.005	0.006

The beta diversity analysis of the resistome reveals distinct clusters between the sludge samples generated by AnMBR and AeMBR. This significant difference in ARGs composition between the sludge samples from the two reactors is confirmed by the ANOSIM test (*R* = 0.99 and *p* = 0.001 for replicate 1; *R* = 0.98 and *p* = 0.001 for replicate 2). Moreover, the influent samples showed greater dissimilarity with the AeMBR sludge than that from the AnMBR (Figure S4). The average *R* coefficient from the ANOSIM between the influent and AeMBR sludge was 0.999, whereas it was only 0.393 for the AnMBR sludge. In terms of ARGs alpha diversity, measured by the Shannon Index, the average values were 5.1 for AnMBR sludge and 5.3 for AeMBR sludge. However, for both replicate runs, there was no statistically significant difference in the index values between the AnMBR and AeMBR sludge (*p* > 0.05).

### 3.3 Proportion of Potential HGT of ARGs in Sludge

There are notable differences in ARGs beta diversity and a lower estimated abundance of ARGs in the AnMBR sludge compared to the AeMBR sludge. To further investigate the potential for ARGs dissemination through HGT, we assessed the normalized average occurrences of potential HGT events in both types of sludge. Our findings revealed that the AeMBR sludge exhibited a higher average of normalized potential HGT events than the AnMBR sludge, consistent across two replicate runs (*p* = 0.0008 and 0.003 for replicates 1 and 2, respectively) (Table 2).

**Table 2:** Average Normalized Total Potential Horizontal Gene Transfer Events and Potential Transfer Events Involving ARGs in the Sludge Derived from AnMBR and AeMBR

parameter	replicate 1 (AnMBR HRT 10 h–AeMBR HRT 8 h)		replicate 2 (AnMBR–AeMBR HRT 8 h)	
	AnMBR sludge	AeMBR sludge	AnMBR sludge	AeMBR sludge
potential HGT ratio	0.6 ± 0.5	2.4 ± 1.1	0.9 ± 0.4	1.6 ± 0.3
potential HGT ratio *p*-value	0.0008		0.003	
potential HGT of ARGs ratio	0.033 ± 0.004	0.067 ± 0.004	0.044 ± 0.0041	0.055 ± 0.005
potential HGT of ARGs *p*-value	0.04		0.047	
potential HGT of ARGs to pathogens ratio	0.006 ± 0.005	0.027 ± 0.006	0.009 ± 0.004	0.021 ± 0.002
potential HGT of ARGs to pathogens *p*-value	0.025		0.045	
Spearman’s ρ (HGT ratio vs MLSS)	0.22 *p*-value > 0.05	0.61 *p*-value = 0.001	0.24 *p*-value > 0.05	0.26 *p*-value > 0.05
Spearman’s ρ (HGT ratio of ARG vs MLSS)	0.08 *p*-value > 0.05	0.7 *p*-value = 0.02	0.18 *p*-value > 0.05	0.12 *p*-value > 0.05

Furthermore, the proportion of potential HGT events related to ARGs transfer was significantly greater in the AeMBR sludge (*p* = 0.04 and 0.047 for replicates 1 and 2, respectively). Additionally, HGT events involving potential pathogens as recipients were significantly more frequent in the AeMBR sludge than in the AnMBR sludge (*p* = 0.025 and 0.045 for replicates 1 and 2, respectively; Table 2).

A significant correlation was observed between the average normalized ratio of potential HGT in the AeMBR sludge and its MLSS content for the first replicate, which averaged 15,377 mg/L (Spearman’s rank correlation coefficient [ρ] = 0.61, *p* = 0.001). This correlation aligns with the relationship between MLSS content and cell density, in which a high amount of biomass generally favors the occurrence of HGT events. Additionally, a notable positive correlation was found between the ratio of potential HGT involving the transfer of ARGs and the MLSS content in the AeMBR sludge (ρ = 0.7, *p* = 0.02). However, significant correlations between potential HGT events in sludge from the AnMBR and its MLSS were not detected in either of the two replicate runs (*p* > 0.05; Table 2).

**Figure 4:**
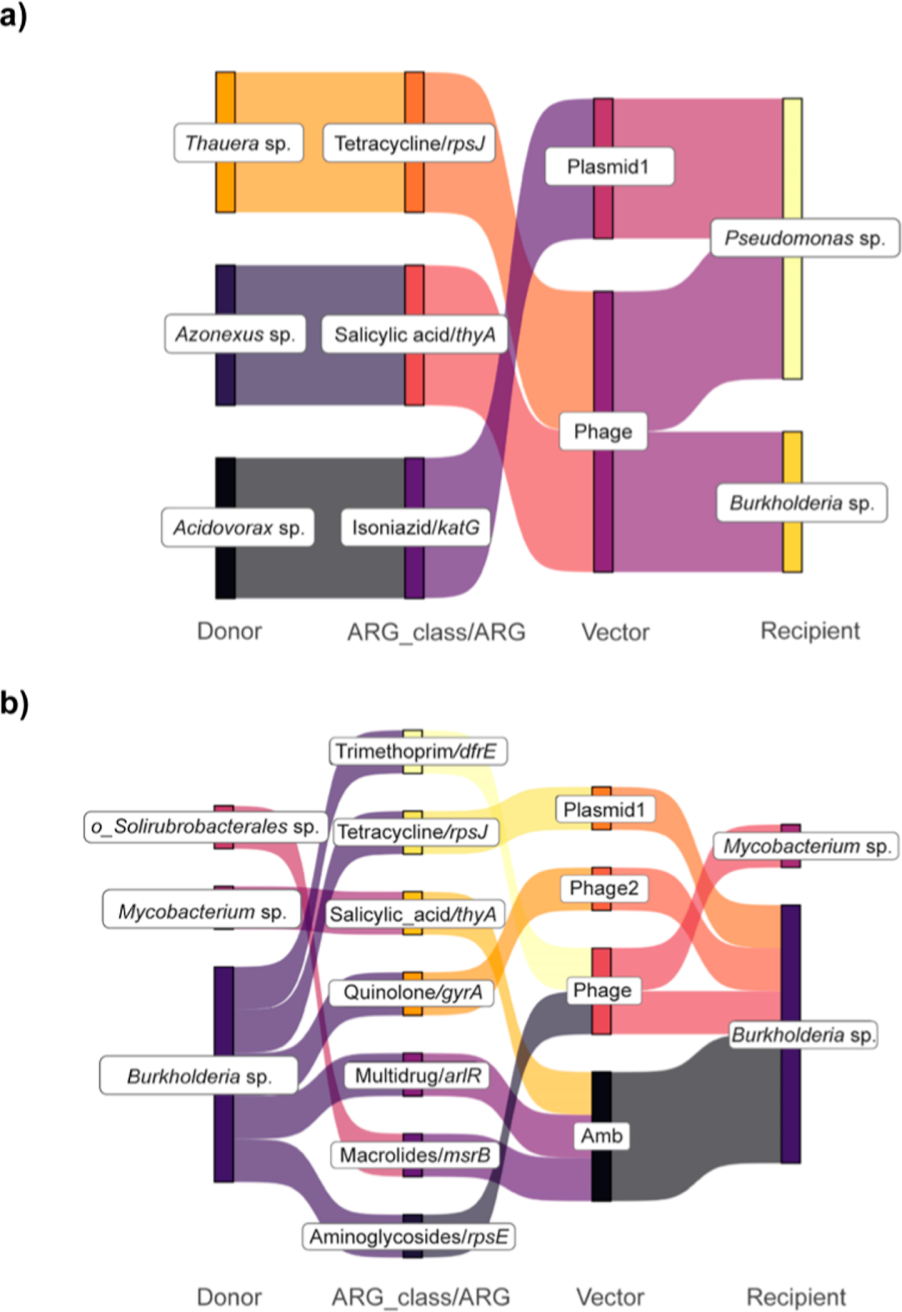
Antimicrobial resistance profiles associated with the (a) AnMBR and (b) AeMBR sludge. Potential HGT of ARGs and vector signature (plasmid/virus) were obtained for the AnMBR and the AeMBR sludge, respectively, whereby the recipients are bacterial genera that contain opportunistic pathogens. Amb stands for ambiguous annotation.

The diversity of ARGs transferred in the AnMBR sludge is lower than that in the AeMBR sludge. For example, the types of ARGs associated with potential HGT events in the AnMBR sludge are primarily involves in resistance to isoniazid, tetracycline, and salicylic acid, with *Pseudomonas* sp. being the main recipient (Figure 4a). By contrast, the AeMBR sludge shows a broader range of HGT events involving various ARGs that confer resistance to quinolones, multidrug, macrolides, trimethoprim, aminoglycosides, salicylic acid, and tetracycline. Herein, the recipients are predominantly different members of Burkholderiales (Figure 4b). Thus, the potential of the HGT of ARGs is significantly lower in the AnMBR sludge than in the AeMBR sludge.

### 3.4 Linking Potential HGT of ARGs in the Sludge of the AeMBR and AnMBR to Phages or Plasmids

The potential for HGT of ARGs in the AnMBR sludge is lower than that in AeMBR sludge, prompting further investigation into HGT events in both aerobic and anaerobic sludge. This analysis aimed to determine whether the dissemination of ARGs is linked to conjugation or transduction. Sequence similarities were noticed between transferred ARGs and viral or plasmid contigs, suggesting possible connections to transduction or conjugation. Cases of no sequence similarity to either viral or plasmid contigs—or similarities to both—were classified as ambiguous or lacking linkage.

For the HGT events of ARGs associated with viral contigs, the average proportion of HGT events was higher in the AnMBR sludge compared with that in the AeMBR sludge across both replicates (averaging 0.24 versus 0.11 and 0.46 versus 0.05 for replicates 1 and 2, respectively; Table 3). However, the differences in the number of HGT events between the AnMBR and AeMBR sludge were not statistically significant (*p* > 0.05; Table 3). Additionally, there was no significant difference in the relative abundance of viral contigs in the AnMBR and AeMBR sludge (Figure S5).

**Table 3:** Proportion of HGT Events Linked to Viral and Plasmid Contigs in the AnMBR and the AeMBR and Number of Provirus and Plasmid Contigs with Conjugation Genes

parameter	replicate 1 (AnMBR HRT 10 h–AeMBR HRT 8 h)		replicate 2 (AnMBR–AeMBR HRT 8 h)	
	AnMBR sludge	AeMBR sludge	AnMBR sludge	AeMBR sludge
proportion of HGT linked to viral contigs	0.24 ± 0.38	0.11 ± 0.14	0.46 ± 0.43	0.05 ± 0.13
proportion of HGT linked to viral contigs *p*-value	*p*-value > 0.05		*p*-value > 0.05	
proportion of HGT linked to plasmid contigs	0.18 ± 0.35	0.33 ± 0.2	0.12 ± 0.21	0.21 ± 0.28
proportion of HGT linked to plasmid contigs *p*-value	*p*-value > 0.05		*p*-value > 0.05	
proportion with no linkage found/ambiguous	0.14 ± 0.33	0.45 ± 0.29	0.13 ± 0.25	0.74 ± 0.38
proportion with no linkage found/ambiguous *p*-value	0.02		0.024	
number of plasmid contigs with full conjugation system/total plasmids linked to HGT of ARGs	1/6	0/7	0/5	0/3
number of provirus contigs/total virus linked to HGT of ARGs	1/11	0/8	1/12	0/4

The number of HGT events of ARGs linked to plasmid contigs was higher in the AeMBR sludge than that in the AnMBR sludge (0.33 versus 0.18 and 0.21 versus 0.12 for replicates 1 and 2, respectively). However, when analyzing the average number of events, there was no significant difference between the AeMBR and AnMBR sludge in either replicate (*p* > 0.05). In cases where HGT events did not show a clear linkage or yielded ambiguous results, the AeMBR sludge exhibited a significantly higher number than the AnMBR sludge, with *p*-values of 0.02 and 0.024 for replicates 1 and 2, respectively (Table 3). Furthermore, only one HGT event involving ARGs transfer was associated with a prophage (Figure S6). This is consistent with the high prevalence of lytic phages present in AnMBR and AeMBR, which showed a relative abundance of 98% (Table S4) compared with lysogenic viral contigs. All identified viral contigs belonged to the order Caudovirales (Table S5). Thus, no significant differences were observed in the number of transferred ARGs contigs related to viral or plasmid contigs between the AnMBR and AeMBR sludge across the two experiments.

However, the abundance of genes related to MGEs indicates that the AnMBR sludge has a lower average abundance of genes associated with conjugation compared with the AeMBR sludge (1.1 × 10^–4^ vs 1.3 × 10^–4^ gene copies per 16S rRNA gene for the AnMBR vs the AeMBR, respectively; Figures S7 and S8).

### 3.5 AnMBR Sludge Supernatant Provides Unfavorable Conditions for Natural Transformation

**Figure 5:**
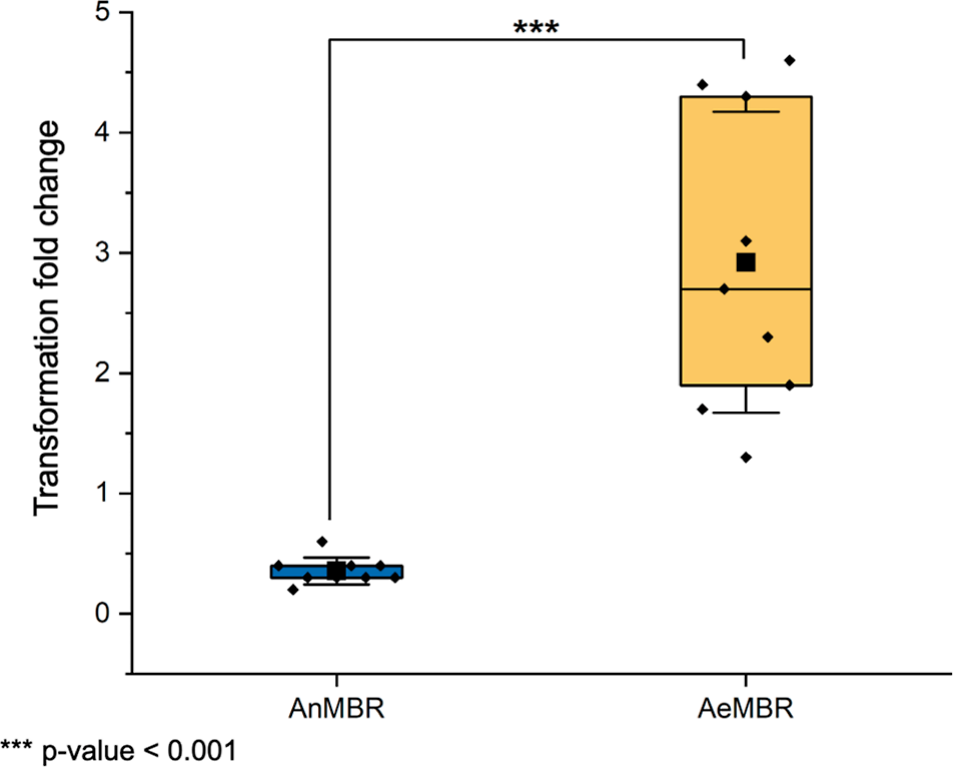
Impact of the supernatant of the sludge of the AnMBR and the AeMBR in the natural transformation of the reporter strain *A. baylyi* ADP1, *** corresponds to *p*-value lower than 0.001.

As the previous metagenomics analysis did not enable easy identification of genetic signatures related to transformation, a complementary laboratory test was performed to further evaluate whether the different conditions of the aerobic and anaerobic sludge could impact the prevalence of natural transformation. With the supernatant of the AnMBR sludge, the transformation rate decreased by 0.3 fold on average compared with that of the control (with a transformation frequency of the AnMBR sludge of 1 × 10^–5^ ± 2.6 × 10^–6^). By contrast, the supernatant of the AeMBR increased the transformation rates by 2.9 fold compared with that of the control, resulting in a transformation frequency of 8.2 × 10^–5^ ± 1.9 × 10^–5^. Hence, there was a significantly lower transformation fold change in *A. baylyi* ADP1 when it was exposed to the supernatant of the AnMBR sludge (*p* < 0.001; Figure 5).

## 4 Discussion

Herein, we assessed the extent of AMR concerns associated with the sludge produced by AnMBR and AeMBR systems, both of which were used to treat the same municipal wastewater. The study aimed to compare these systems in terms of their contribution to ARGs dissemination due to sludge disposal. Our findings suggest that using AnMBR to treat wastewater is more favorable for reducing AMR concerns than the AeMBR owing to a lower sludge volume of the former and, consequently, a lower abundance of ARGs associated with the sludge disposal process. These results are consistent with those of a previous study where the ARGs abundance was reported to be lower in the sludge and effluent of an AnMBR than those of an AeMBR, both of which were fed with synthetic wastewater supplemented with antibiotics.^44^ However, the primary limitation of this study is that the experimental design only included eight sampling points collected over 2 months, which may not reflect long-term trends. Nevertheless, the key findings elucidated in this study—specifically that AnMBR technology can mitigate risks associated with ARGs relatively better than AeMBR technology—were observed reproducibly in the two biologically independent runs.

Although there are processes to stabilize aerobic sludge before disposal (i.e., either through anaerobic or aerobic digestion), previous studies have found that certain types of ARGs, which encode resistance to tetracyclines, sulfonamides, macrolides, chloramphenicol, aminoglycosides and beta-lactams in the stabilized sludge, are enriched or remain constant following the digestion process (*tetG*, *sul1*, *mefA*, *ermB*, *catb3*, *aadA*, and *bla*_*OXA-1*_).^45−47^ In particular, *sul1* has a high mobility rate and has been previously correlated with MGE *int1*, while multidrug resistance genes have been reported as AMRs of concern,^48^ particularly aminoglycosides, which have been linked to bacterial priority pathogens in the 2024 WHO report.^49^ Thus, there is a risk associated with disposing sludge even after stabilization, and one preventative strategy is to reduce the volume of sludge being disposed. With this consideration, anaerobic wastewater treatment, which generates a lower sludge volume, is particularly advantageous compared with AeMBR in minimizing the extent of AMR dissemination from sludge waste.

In addition to a lower risk of ARGs dissemination related to sludge volume, our findings suggest that using AnMBR to treat wastewater results in the production of sludge with a lower potential for HGT events, particularly those associated with ARGs. These HGT events occur through three main mechanisms: conjugation, transduction, and transformation. For HGT through conjugation, a higher abundance of MGE was found in the aerobic versus anaerobic configuration (Figures S7 and S8). This observation aligns with an earlier study that reported a lower decay of *Escherichia coli* PI7 and *bla*_NDM-1_ in anaerobic sludge on a bench scale, which also noted that no HGT events of conjugation by cultivation methods were reported for the anaerobic treatment.^18^ By contrast, the earlier study recovered viable transconjugants from the aerobic sludge.

Conversely, this study found a significantly higher frequency of natural transformation for AeMBR sludge (Figure 5). Several physical and chemical factors that cause stress in the bacterial population and lead to cell lysis can influence the occurrence of transformation, one of which is the generation of reactive oxygen species, which can facilitate transformation.^43^ Lytic phage activity could also play a role, releasing extracellular DNA that might contain ARGs.^50,51^ As extracellular ARGs are released due to cell lysis, some of this DNA can be attached to extracellular polymeric substances, which was found to have a higher abundance than cell-free extracellular ARGs in activated sludge.^52^ Considering the positive correlation between HGT and MLSS in aerobic sludge and the routine maintenance of these systems at a higher MLSS than in anaerobic sludge, this could explain why there was a higher frequency of natural transformation in AeMBR sludge than in AnMBR sludge.

In the context of HGT through transduction, no significant difference was observed in the proportion of HGT events for ARGs transfer between AnMBR and AeMBR. The role of bacteriophages in the transfer of ARGs has received limited attention, making it unclear how much transduction events contribute to ARGs dynamics in various wastewater treatment systems.^47^ One factor that could influence phage activity is the redox conditions present in these systems. Prior research has suggested that the presence of oxidants may reduce the production of infective MS2 phages, and studies have shown minimal induction of temperate phages,^49^ with no significant prophage induction events detected in activated sludge.^50^ In anaerobic digestion systems, earlier studies indicated that viral OTUs associated with ARGs contribute only a small fraction (0.57%) to the overall resistome. Furthermore, it appears that phages are more involved in the lysis of ARB rather than facilitating transduction in these anaerobic environments.^51^

This study found no significant differences in the role of transduction for HGT in aerobic and anaerobic sludge, and only one prophage was identified in one of the replicates for the AnMBR, which belonged to the Caudovirales. Viruses from the order Caudovirales, specifically from the families Siphoviridae and Myoviridae, have been associated with putative ARGs transfer.^53^ Currently, there are limitations related to the efficient recovery and concentration of viruses from water samples and a low taxonomical resolution due to the vast majority of viruses that have yet to be comprehensively identified from various sequencing efforts. Thus, further long-term studies are needed to gain more insight into the prevalence of ARGs in phages and how operational factors can influence these transduction events and their associated risks.

Another aspect to consider is the diversity of ARGs transferred between the AnMBR and the AeMBR. There are significant differences in the ARGs beta diversity between the AnMBR and AeMBR sludge, with the latter having more types of ARGs transferred to potential bacterial recipients. These differences could be partly attributed to the dissimilarity in the microbial community profiles of the AnMBR and AeMBR sludge. For example, in the AnMBR sludge, the prevalence of members from the phyla Proteobacteria and Bacteroidetes has been previously reported in AnMBRs, especially in mesophilic systems.^44,54^ There is a higher similarity in the beta diversity of the microbial community/ARGs between the influent and AnMBR sludge compared with that between influent and AeMBR sludge. This could be related to similar conditions regarding redox potential in the influent and the anaerobic fermenter. By contrast, the persistence of Anaerolineae in the sludge of the AeMBR is related to the configuration of this plant, which has anoxic/oxic-activated sludge located before the membrane tank.^55^ This bacterial class has been reported previously in anoxic–oxic and anaerobic–anoxic–oxic systems and is associated with sludge floc stabilization.^56^ Lower relative abundance of Anaerolineae was also found in the sludge of AnMBR, which is similar as reports in anaerobic systems for the treatment of municipal wastewater.^57^

Environmental conditions affect the microbial community profile, and the populations that thrive in a particular environment will influence the transmission of ARGs through vertical transfer.^48^ Alternatively, a higher abundance of transferable MGEs, physical or chemical factors, and higher cell density in the aerobic configuration could favor a greater potential for HGT, making it more likely to transfer a wider diversity of ARGs.

In summary, these results show a significantly lower absolute abundance of ARGs and potential for HGT events involving ARGs in anaerobic sludge than in aerobic sludge. By contrast, a higher diversity of ARGs is transferred in aerobic sludge. These findings suggest that in addition to the advantages of anaerobic systems—specifically lower energy consumption, energy generation from biogas production, and lower sludge waste disposal—anaerobic systems can contribute less toward the dissemination of AMR in their solid waste.

## Supporting Information Available

Supplementary methods that denote the calculation of the abundance of ARGs with scale factor of sludge disposal, details on the method used by MetaCHIP for the identification of HGT events, additional methods for the calculation of abundance of virus and plasmids, additional details on the strain of *A. baylyi* ADP1 and statistical analysis used in this study; supplementary tables that denote the water quality parameters and performance of AnMBR and AeMBR for replicate 1 and 2, sampling dates and filtered total DNA reads, percentage of viral contigs corresponding to provirus and lytic virus in the AnMBR and AeMBR sludge for the two replicates, most abundant virus taxa in order and family level in the AnMBR and AeMBR sludge for the two replicates; and supplementary figures that denote log removal value of bacterial cells in AnMBR and AeMBR, proportion of viral contig reads in AnMBR and AeMBR sludge and HGT transfer of ARGs linked to provirus (PDF)
