# Supplementary material for: Metagenomic Insights in Antimicrobial Resistance Threats in Sludge from Aerobic and Anaerobic Membrane Bioreactors
Source: Environ Sci Technol. 2025 Mar 12;59(11):5636–46. doi: 10.1021/acs.est.4c10879 (PMC11948473; doi:10.1021/acs.est.4c10879)
Supplement: Supplementary file 1 — es4c10879_si_001.pdf [file es4c10879_si_001.pdf]

Supporting information

Metagenomic insights in antimicrobial resistance threats in sludge from aerobic and anaerobic membrane bioreactors

Julie Sanchez Medina[1], Shuo Zhang[2], Shaman Narayanasamy[2], Changzhi Wang[3], Bothayna Al-Gashgari[4], Pei-Ying Hong[1,2,3,4].

1. Environmental Science and Engineering Program, Biological and Environmental Science and Engineering Division, King Abdullah University of Science and Technology (KAUST), Thuwal 23955-6900, Kingdom of Saudi Arabia

2. Center of Excellence on Sustainable Food Security, King Abdullah University of Science and Technology (KAUST), Thuwal, Saudi Arabia

3. Bioengineering Program, Biological and Environmental Science and Engineering Division, King Abdullah University of Science and Technology (KAUST), Thuwal 23955-6900, Kingdom of Saudi Arabia

4. Bioscience Program, Biological and Environmental Science and Engineering Division, King Abdullah University of Science and Technology (KAUST), Thuwal 23955-6900, Kingdom of Saudi Arabia

*Corresponding author:
Pei-Ying Hong peiying.hong@kaust.edu.sa

Summary of contents

Number of supplementary methods: 5

Number of supplementary figures: 8

Number of supplementary tables: 6

Table of Contents

Text S1. Calculation of ARG abundance with scale factor of sludge disposal per treated volume3

Text S2. Criteria of MetaCHIP workflow...4

Text S3. Additional methods of omics-based analysis to characterize HGT related to transduction and conjugation...4

Text S4. Characteristics of the reporter strain of *A. baylyi* ADP1 5

Text S5. Statistical analysis...5

Table S1. Water quality parameters and performance of AnMBR vs AnMBR replicate 1............6

Table S2. Water quality parameters and performance of AeMBR vs AnMBR replicate 2............6

Table S3. Average proportion of ARG and abundance per sludge volume disposed for AnMBR sludge vs AeMBR...7

Table S4. Sampling dates and filtered total DNA reads. 7

Table S5. Percentage of the total clustered viral contigs corresponding to provirus and lytic........8

virus in the sludge of AnMBR and AeMBR for the two replicates...8

Table S6. Most abundant virus taxa in order and family level from total clustered viral 8

contigs in the sludge of AnMBR and AeMBR for the two replicates 8

Figure S1. Overview of experimental design. Both AnMBR and AeMBR were operated with the same municipal wastewater as influent. Weekly sampling was conducted at the points denoted with star, and DNA extraction of influent and sludge from both reactors and metagenomic analysis were performed. 9

Figure S2. Log removal value of bacterial cells for AnMBR and AeMBR reactors during the duration of the study. 10

Figure S3. Bootstrapped metric multi-dimensional scaling plot for relative abundance of bacteria at genus level in both influent and sludge samples from AnMBR and AeMBR. Square-root transformation was performed and resemblance matrix was constructed based on Bray-Curtis. 95 % confidence-level bootstrap regions are shown. 11

Figure S4. Bootstrapped metric multi-dimensional scaling plot for relative abundance of ARG in influent and sludge from AnMBR and AeMBR. Square-root transformation was performed and resemblance matrix was constructed based on Bray-Curtis. 95 % confidence-level bootstrap regions are shown. 12

Figure S5. Proportion of viral contigs reads for AnMBR and AeMBR sludge 13

Figure S6. HGT transfer of ARG mediated by provirus in AnMBR sludge, G corresponds to genes and *basR* is the transferred ARG...14

Figure S7. Gene copies per 16S rRNA genes for the major categories of mobile genetic elements for the sludge of the AeMBR. Replicate 1 comprises week 1 to 8 (W1-W8) while replicate 2 comprises week 9 to 16 (W9-W16). 15

Figure S8. Gene copies per 16S rRNA gene for the major categories of mobile genetic elements for the sludge of the AnMBR. Replicate 1 comprises week 1 to 8 (W1-W8) while replicate 2 comprises week 9 to 16 (W9-W16). 15

Text S1. Calculation of ARG abundance with scale factor of sludge disposal per treated volume

For the calculation of ARG abundance in terms of copy number the following formula. First, the equation used is considered the conversion from total DNA concentration to copy number using the Avogadro number, the length of the reads and the weight of 1bp of DNA. If this is multiplied by the proportion of ARG reads over the total DNA reads, we will obtain the ARG abundance in terms of copy number per µl.

Subsequently, considering the volume of extracted DNA and the mass of sludge used for the DNA extraction (obtained from the MLSS and the volume of sludge extracted), we will obtain the ARG abundance in terms of copy number per gram of sludge. Then as we know the grams of sludge disposed per volume of treated water, we use that factor to obtain the estimated ARG abundance in terms of copy number per m^3 of treated water.

The abundance in terms of ARG copies per volume of DNA extracted is calculated

$$ARG\ abundance\ (copy\ number) = ARGratio\ x \frac{Conc_{DNA}(\frac{ng}{\mu L})xN_A}{length\ (bp)x\frac{1x10^9 g}{1ng}x660(\frac{g/mol}{bp})}$$

$$N_A = Avogadro\ number\ (6.022x10^{23})$$

$$Weight\ of\ 1bp\ of\ DNA = 660\ g/mol$$

Then as 30 μL was the extracted volume of DNA, this factor is used along with the MLSS of the sludge (in g/L) and the original volume of sludge used to extract DNA (50mL) to calculate the copy number per gram of sludge

$$ARG\ abundance\ (copy\ per\ g\ of\ sludge\ ) = \frac{ARG\ abundance\ \left(\frac{copy\ number}{\mu L}\right) x 30\mu L}{MLSS\ sludge\ (\frac{g}{L}) x 0.05L}$$

Next the sludge disposal is calculated per volume of treated water in dry basis assuming dewatering and thickening (20% solids content)

$$ARG\ abundance\ \left(\frac{copy\ number}{m^3\ of\ treated\ water}\right) = ARG\ abundance\ \left(\frac{copy\ number}{g\ of\ sludge}\right) x \frac{g\ of\ sludge\ disposed}{m^3\ of\ treated\ water}$$

Text S2. Criteria of MetaCHIP workflow

This analysis is based on sequence similarity of genes that could potentially be transferred. A phylogenetic analysis of these aforementioned genes was conducted where the transferred genes tree is compared against the phylogenetic tree based on single-copy genes (SCGs). Then reconciliation of the two gene and species trees and donor and recipient identification is performed with ranger-DTL[1].

Text S3. Additional methods of omics-based analysis to characterize HGT related to transduction and conjugation

To compare the abundance of viral and plasmid contigs present in the AnMBR and AeMBR sludge for both replicates, CD-HIT with default settings was used to obtain a clustered set of the viral and plasmid contigs[43] and CoverM was used to calculate the abundance of viral and

plasmid contigs with the clustered set used as reference for the contigs assembly of each sample. In addition, the ORFs were obtained from Prodigal [44]

Text S4. Characteristics of the reporter strain of *A. baylyi* ADP1

This reporter strain lacks a functional promoter for the expression of spectinomycin resistance gene in the absence of natural transformation. The functional promoter is introduced as an extracellular DNA, which when successfully translocated through the cell wall membrane of *A. baylyi* ADP1 and undergo homologous recombination at the intended position in the chromosomal genome, will result in the expression of spectinomycin resistance, thus indicating the presence of natural transformation[2].

Text S5. Statistical analysis

Levene test was used to evaluate if there is a significant difference in the variance for the relative abundance of ARG in both systems. An ANOSIM value of 1 suggests complete dissimilarity between compared groups while a value close to 0 suggests high similarity between compared groups. Spearman's rank correlation coefficient test was performed to evaluate if there was association between the relative abundance of ARG types vs the relative abundance of MGE gene categories in both AnMBR and AeMBR. Mann-Whitney analysis was performed to evaluate the difference in average proportion of potential HGT, the proportion of HGT where ARG was transferred and the proportion of HGT events where an opportunistic pathogen acts as the recipient and the proportion of HGT events of ARG transfer linked to plasmid or viral contigs.

Table S1. Water quality parameters and performance of AnMBR vs AnMBR replicate 1

Replicate 1 (AnMBR HRT 10h/ MBR HRT 8h)		
**Parameter**	**AnMBR**	**AeMBR**
**COD removal (%)**	89±2	92.1±2
**Nitrate in effluent (mg/L)**	0.018±0.002	0.026±0.012
**Nitrite in effluent (mg/L)**	0.26±0.02	8.87±4.01
**Phosphate in effluent (mg/L)**	2.60±0.24	3.40±1.3
**Ammonia in effluent (mg/L)**	18.5±1.4	<2
**Bacterial cells LRV (mg/L)**	2±0.19	2.12±0.4
**Biogas production (LCH4/ day)**	1.48±0.2	-----------

Table S2. Water quality parameters and performance of AeMBR vs AnMBR replicate 2

Replicate 2 (AnMBR HRT 8h/ MBR HRT 8h)		
**Parameter**	**AnMBR**	**AeMBR**
**COD removal (%)**	86.1±3	91.8±1.7
**Nitrate in effluent (mg/L)**	0.019±0.001	0.151±0.12
**Nitrite in effluent (mg/L)**	0.27±0.03	8.1±2.75
**Phosphate in effluent (mg/L)**	3.30±1.13	3.70±1.05
**Ammonia in effluent (mg/L)**	21.36±2.54	<2
**Bacterial cells LRV (mg/L)**	2.26±0.4	2.35±0.45
**Biogas production (LCH4/ day)**	1.58+0.4	--------------

Table S3. Average proportion of ARG and abundance per sludge volume disposed for AnMBR sludge vs AeMBR

Parameter	Replicate 1 (AnMBR HRT 10h-AeMBR HRT 8h)		Replicate 2 (AnMBR-AeMBR HRT 8h)	
	**AnMBR sludge**	**AeMBR sludge**	**AnMBR sludge**	**AeMBR sludge**
**Average proportion ARG (%)**	$6.3\times10^{-3} \pm 2.9\times10^{-3}$	$5.5\times10^{-4} \pm 1.5\times10^{-4}$	$1.2\times10^{-2} \pm 8.2\times10^{-2}$	$3.8\times10^{-4} \pm 1\times10^{-4}$
**ARG abundance scaled by disposed sludge (copies /m³ of treated water)**	$2.\times10^{5} \pm 1.5\times10^{5}$	$4.6\times10^{8} \pm 2.9\times10^{8}$	$4.2\times10^{5} \pm 1.3\times10^{5}$	$3.2\times10^{8} \pm 1.7\times10^{8}$

Table S4. Sampling dates and filtered total DNA reads.

Sample-date (year 2021)	Replicate	HRT AnMBR	HRT AeMBR	AnMBR sludge total reads	AeMBR sludge total reads
**July 7**	1	10h	8h	37456706	70088584
**July 14**	1	10h	8h	39901210	74445216
**July 26**	1	10h	8h	55034122	76437992
**August 3**	1	10h	8h	63850590	52553258
**August 9**	1	10h	8h	54301850	60926202
**August 16**	1	10h	8h	37062758	77948294
**August 23**	1	10h	8h	58466078	65741986
**August 29**	1	10h	8h	49429516	71291400
**September 5**	1	8h	8h	46597632	64991794
**September 12**	2	8h	8h	60207198	60818058
**September 19**	2	8h	8h	55780482	78907766
**September 26**	2	8h	8h	55767334	75797880

**October 3**	2	8h	8h	60072706	62239410
**October 17**	2	8h	8h	75001898	67079230
**October 24**	2	8h	8h	73733460	71365754
**October 31**	2	8h	8h	58248432	66041394

Table S5. Percentage of the total clustered viral contigs corresponding to provirus and lytic virus in the sludge of AnMBR and AeMBR for the two replicates

**Parameter**	**Replicate 1 (AnMBR HRT 10h-AeMBR HRT 8h)**		**Replicate 2 (AnMBR-AeMBR HRT 8h)**	
	AnMBR	AeMBR	AnMBR	AeMBR
**Provirus**	2.6%	2.2%	2.5%	2.0%
**Lytic virus**	97.4%	97.8%	97.5%	98.0%

Table S6. Most abundant virus taxa in order and family level from total clustered viral contigs in the sludge of AnMBR and AeMBR for the two replicates

**Parameter**	**Replicate 1 (AnMBR HRT 10h/ MBR HRT 8h)**		**Replicate 2 (AnMBR HRT 8h/ MBR HRT 8h)**	
**Viral order:family**	AnMBR	AeMBR	AnMBR	AeMBR
**Caudoviricetes;**	90.7%	93.0%	90.4%	93.7%
**Caudoviricetes;Ackermannviridae**	0.4%	0.1%	0.3%	0.2%
**Caudoviricetes;Autographiviridae**	0.6%	0.5%	0.9%	0.3%
**Caudoviricetes;Crassvirales**	0.5%	0.5%	0.6%	0.1%
**Caudoviricetes;Demerecviridae**	0.5%	0.1%	0.6%	0.1%
**Caudoviricetes;Herelleviridae**	0.5%	0.1%	0.3%	0.1%
**Caudoviricetes;Schitoviridae**	0.2%	0.0%	0.3%	0.0%
**Caudoviricetes;Straboviridae**	1.3%	0.1%	1.0%	0.2%
**Others**	5.2%	5.7%	5.6%	5.4%

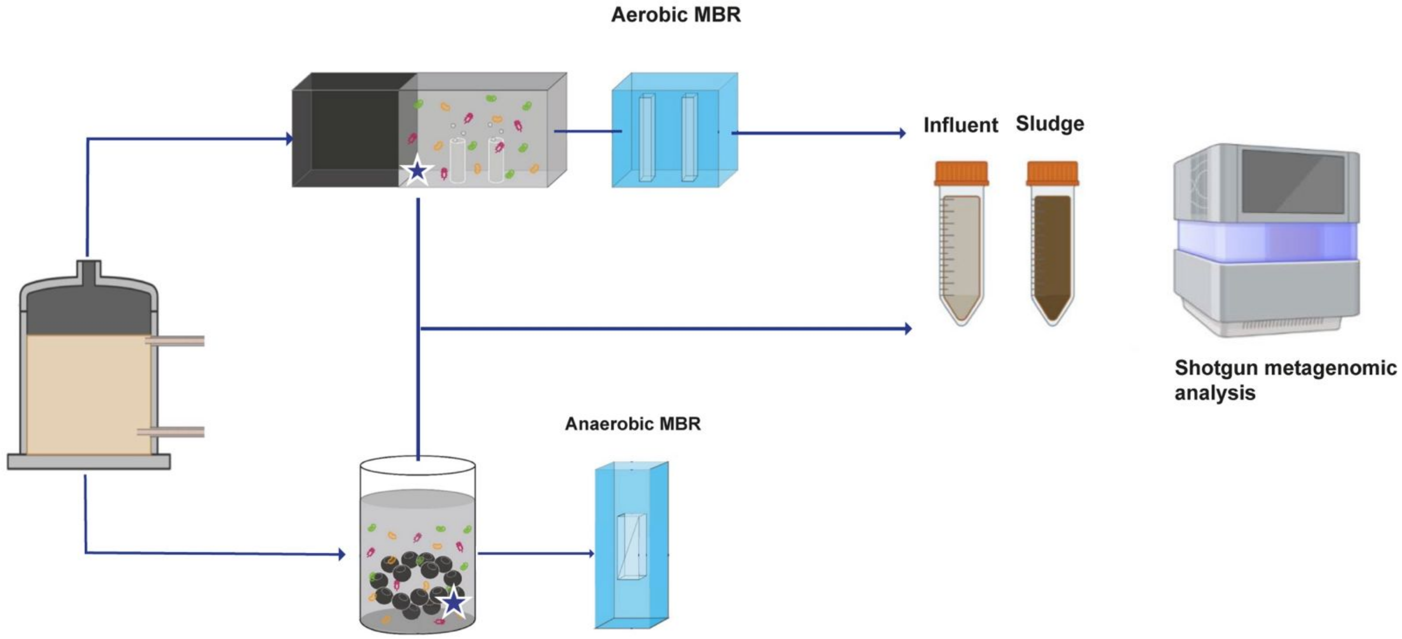

Figure S1. Overview of experimental design. Both AnMBR and AeMBR were operated with the same municipal wastewater as influent. Weekly sampling was conducted at the points denoted with star, and DNA extraction of influent and sludge from both reactors and metagenomic analysis were performed.

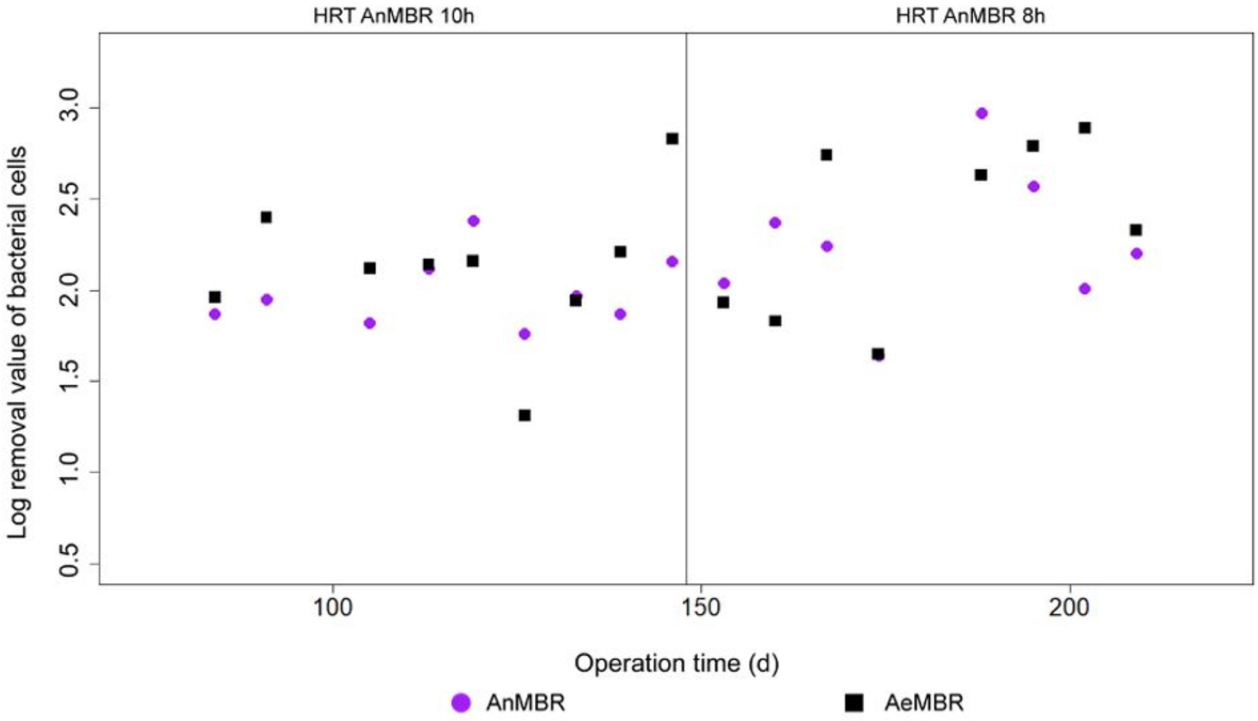

Figure S2. Log removal value of bacterial cells for AnMBR and AeMBR reactors during the duration of the study.

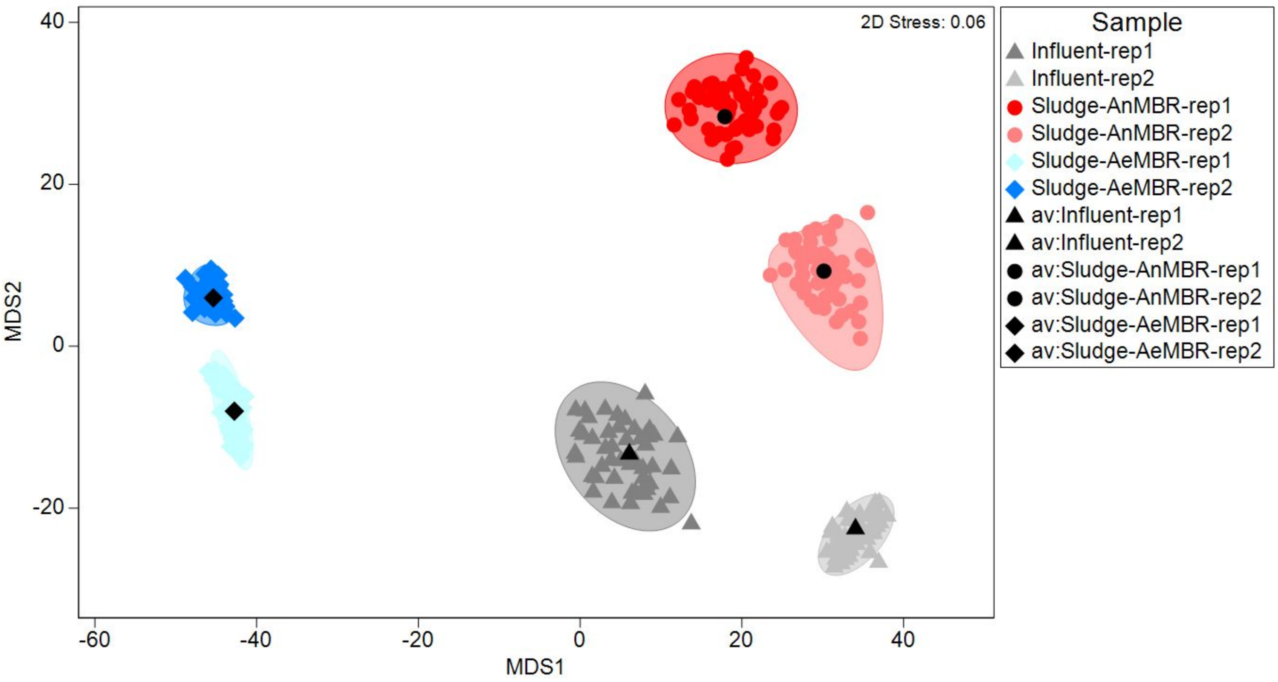

Figure S3. Bootstrapped metric multi-dimensional scaling plot for relative abundance of bacteria at genus level in both influent and sludge samples from AnMBR and AeMBR. Square-root transformation was performed and resemblance matrix was constructed based on Bray-Curtis. 95 % confidence-level bootstrap regions are shown.

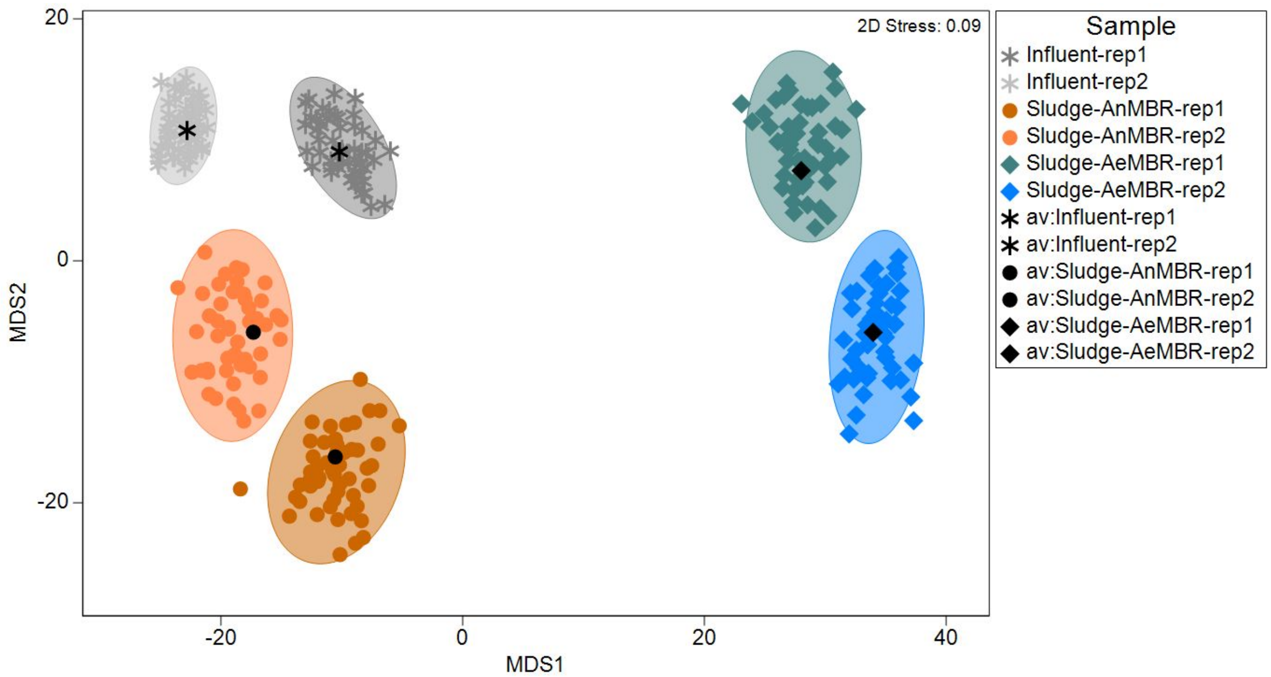

Figure S4. Bootstrapped metric multi-dimensional scaling plot for relative abundance of ARG in influent and sludge from AnMBR and AeMBR. Square-root transformation was performed and resemblance matrix was constructed based on Bray-Curtis. 95 % confidence-level bootstrap regions are shown.

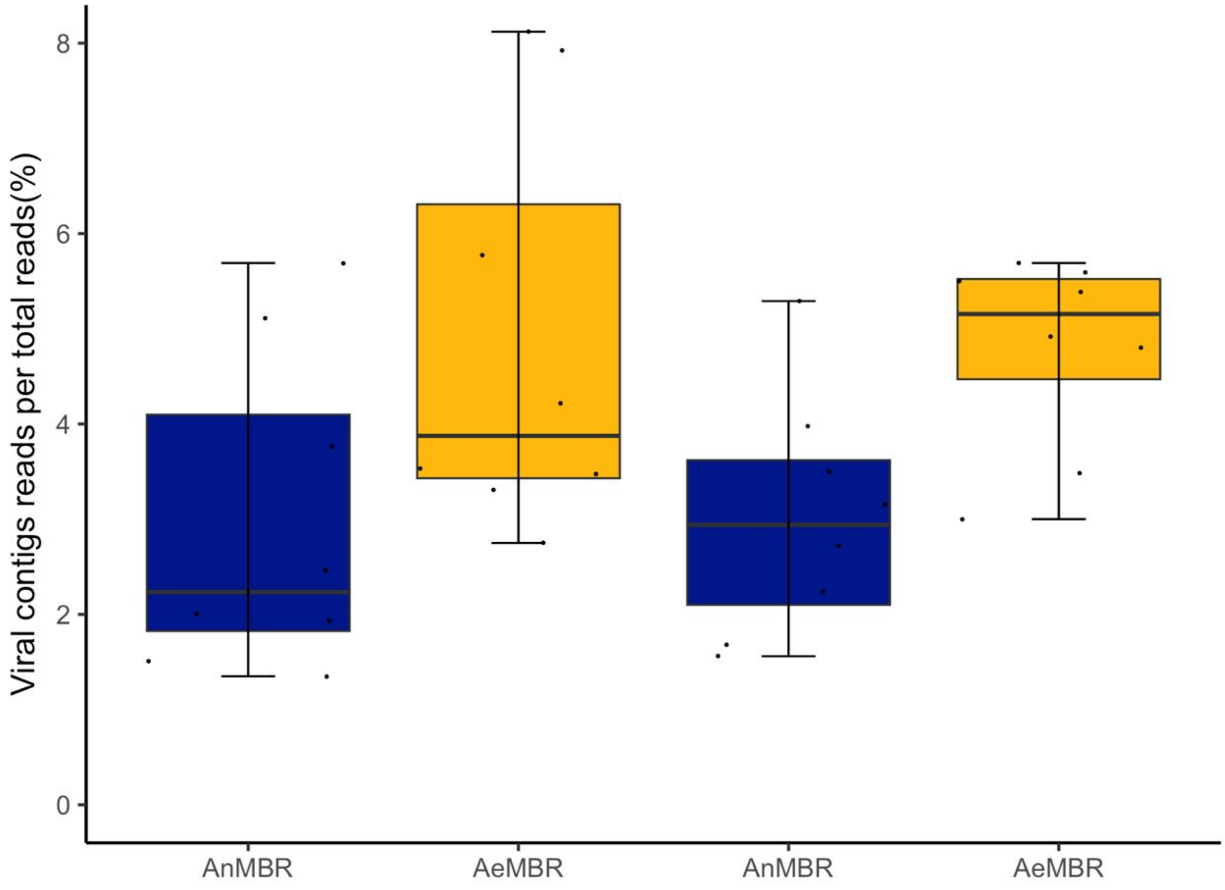

Figure S5. Proportion of viral contigs reads for AnMBR and AeMBR sludge

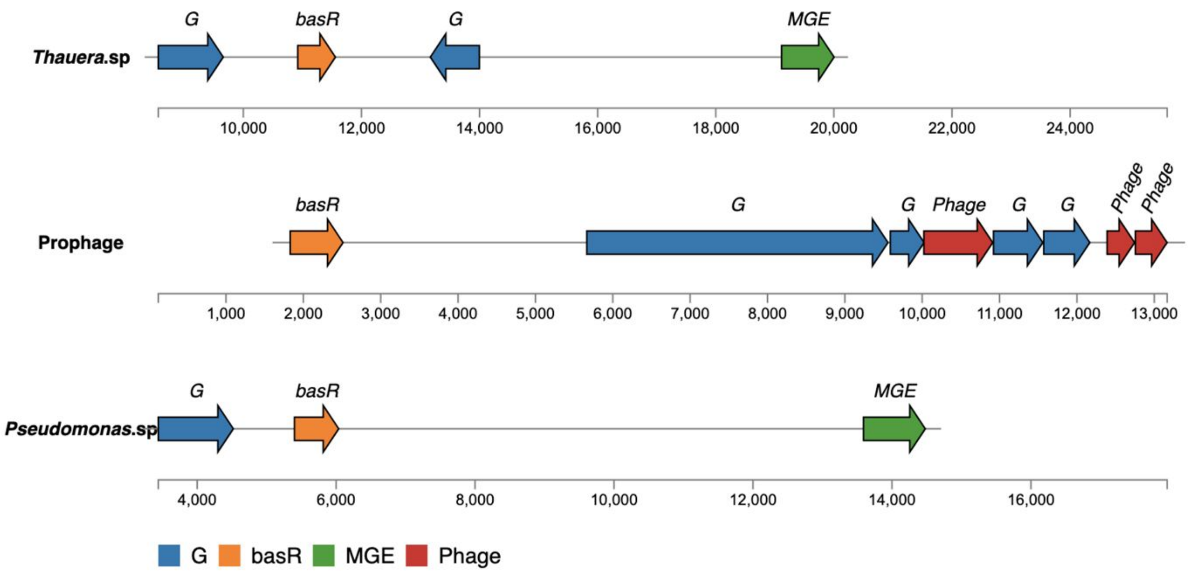

Figure S6. HGT transfer of ARG mediated by provirus in AnMBR sludge, G corresponds to genes and *basR* is the transferred ARG

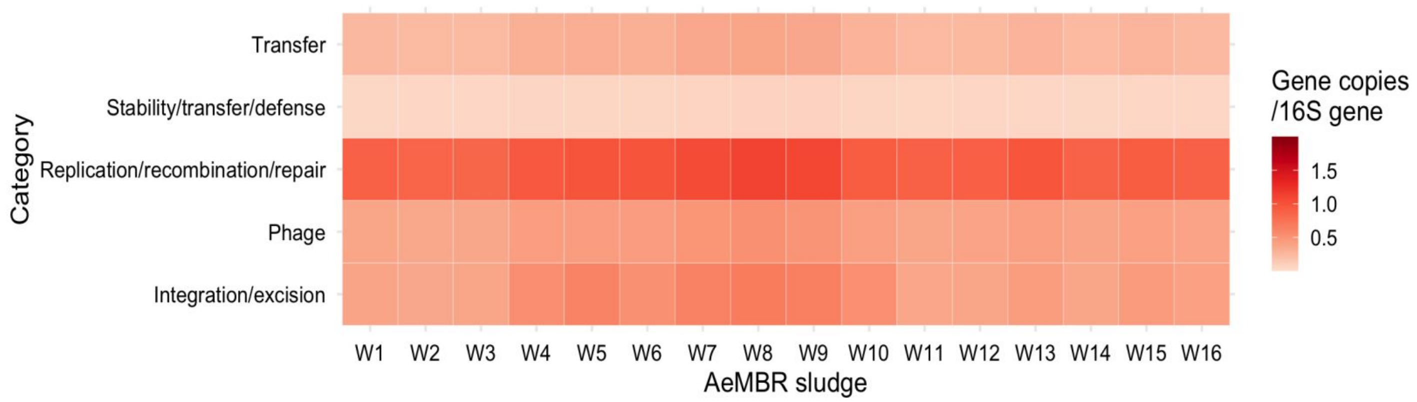

Figure S7. Gene copies per 16S rRNA genes for the major categories of mobile genetic elements for the sludge of the AeMBR. Replicate 1 comprises week 1 to 8 (W1-W8) while replicate 2 comprises week 9 to 16 (W9-W16).

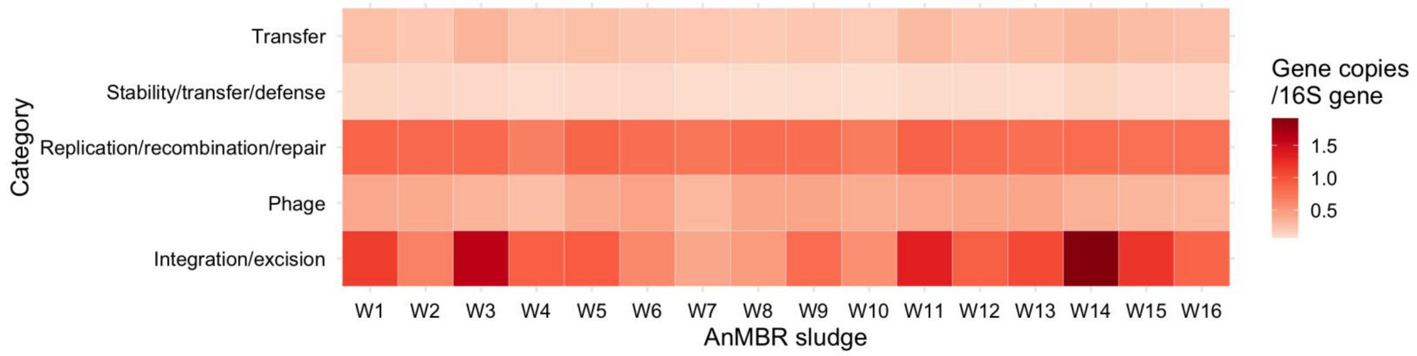

Figure S8. Gene copies per 16S rRNA gene for the major categories of mobile genetic elements for the sludge of the AnMBR. Replicate 1 comprises week 1 to 8 (W1-W8) while replicate 2 comprises week 9 to 16 (W9-W16).

1. Bansal, M. S.; Kellis, M.; Kordi, M.; Kundu, S., RANGER-DTL 2.0: rigorous reconstruction of gene-family evolution by duplication, transfer and loss. *Bioinformatics* **2018,** *34*, (18), 3214-3216.
2. Rizzi, A.; Pontiroli, A.; Brusetti, L.; Borin, S.; Sorlini, C.; Abruzzese, A.; Sacchi, G. A.; Vogel, T. M.; Simonet, P.; Bazzicalupo, M., Strategy for in situ detection of natural transformation-based horizontal gene transfer events. *Applied and environmental microbiology* **2008,** *74*, (4), 1250-1254.
